# Removal of Azo Dyes from Water Using Natural *Luffa cylindrica* as a Non-Conventional Adsorbent

**DOI:** 10.3390/molecules29091954

**Published:** 2024-04-24

**Authors:** Ma. Guadalupe Aranda-Figueroa, Adriana Rodríguez-Torres, Alexis Rodríguez, Gloria Ivette Bolio-López, David Osvaldo Salinas-Sánchez, Dulce Ma. Arias-Atayde, Rosenberg J. Romero, Maria Guadalupe Valladares-Cisneros

**Affiliations:** 1 Facultad de Ciencias Químicas e Ingeniería, Universidad Autónoma del Estado de Morelos, Avenida Universidad 1001, Colonia Chamilpa, Cuernavaca 62209, Mexico; ma.arandafgr@uaem.edu.mx; 2 Departamento de Ingeniería en Aeronáutica, Universidad Politécnica Metropolitana de Hidalgo, Tolcayuca 1009 Ex Hacienda San Javier, Tolcayuca 43860, Mexico; adrodriguez@upmh.edu.mx; 3 Centro de Investigación en Biotecnología, Universidad Autónoma del Estado de Morelos, Avenida Universidad 1001, Colonia Chamilpa, Cuernavaca 62209, Mexico; alexis.rodriguez@uaem.mx; 4 Dirección de Ciencias Básicas e Ingeniería, Universidad Popular de la Chontalpa, Carretera Cardenas-Huimanguillo Km 2.0, Cardenas 86500, Mexico; gloria.bolio@upch.mx; 5 Centro de Investigación en Biodiversidad y Conservación, Universidad Autónoma del Estado de Morelos, Avenida Universidad 1001, Colonia Chamilpa, Cuernavaca 62209, Mexico; davidos@uaem.mx; 6 Centro de Investigación y Educación Ambiental Sierra de Huautla (CEAMISH), Universidad Autónoma del Estado de Morelos, Avenida Universidad 1001, Colonia Chamilpa, Cuernavaca 62209, Mexico; dulce@uaem.mx; 7 Centro de Investigación en Ingeniería y Ciencias Aplicadas, Universidad Autónoma del Estado de Morelos, Avenida Universidad 1001, Colonia Chamilpa, Cuernavaca 62209, Mexico

**Keywords:** *Luffa cylindrica*, unmodified fiber, non-conventional adsorbent, azo dyes, dye removal

## Abstract

Reducing high concentrations of pollutants such as heavy metals, pesticides, drugs, and dyes from water is an emerging necessity. We evaluated the use of *Luffa cylindrica* (*Lc*) as a natural non-conventional adsorbent to remove azo dye mixture (ADM) from water. The capacity of *Lc* at three different doses (2.5, 5.0, and 10.0 g/L) was evaluated using three concentrations of azo dyes (0.125, 0.250, and 0.500 g/L). The removal percent (R%), maximum adsorption capacity (Q_m_), isotherm and kinetics adsorption models, and pH influence were evaluated, and Fourier-transform infrared spectroscopy and scanning electron microscopy were performed. The maximum R% was 70.8% for 10.0 g L^−1^ *Lc* and 0.125 g L^−1^ ADM. The Q_m_ of *Lc* was 161.29 mg g^−1^. Adsorption by *Lc* obeys a Langmuir isotherm and occurs through the pseudo-second-order kinetic model. Statistical analysis showed that the adsorbent dose, the azo dye concentration, and contact time significantly influenced R% and the adsorption capacity. These findings indicate that *Lc* could be used as a natural non-conventional adsorbent to reduce ADM in water, and it has a potential application in the pretreatment of wastewaters.

## 1. Introduction

Processing and manufacturing industries are sources of generated wastewater, which includes a mixture of pollutants, including heavy metals, pesticides, azo dyes, drugs, and oils. Wastewater can be classified depending on the type of compounds it contains. For instance, according to the World Bank, textile dyeing generates between 17% and 20% of industrial wastewater [1]. Mainly, textile wastewater contains dyes and salts and largely aqueous soluble inorganic-colored complexes. Some authors have reported that wastewater from the leather industry may contain up to 200 mg L^−1^ of dyes [2]. 

Most color-based industries commonly employ, at the end of the process, a wastewater treatment to reduce the pollutant concentrations before leaving water in open sources. However, the complexity and heterogeneity of the pollutants in wastewater need highly efficient technologies. Hence, water treatment plants are closely monitored and regulated by standard protocols to meet the required standards for water quality [3]. Higher pollutant concentration disposed in water is correlated with a huge risk and hazardous impacts on the environment and human health [2,4]. Consequently, water treatment is a leading factor in the accomplishment of sustainable water provision [5].

The presence of dyes in water is highly visible. Moreover, even at concentrations of <1.0 mg L^−1^ [6], there can be significant changes in the physicochemical properties of water. For example, dyes reduce the transmission and penetration of sunlight into the water and thus affect the biochemical oxygen demand (BOD), chemical oxygen demand (COD), and pH [7,8]. Synthetic textile dyes are widely used due to their easy application and ability to give clothing brighter and more durable colors [9]. 

Azo dyes are the largest and the most important group of synthetic dyes [10]. They are highly toxic and stable under different environmental conditions (light, heat, etc.) and resist degradation processes (aerobic digestion and oxidizing agents) [11]. 

Several technologies are being studied as part of wastewater treatment, including the combination of pre-treatments and primary treatments to reduce the organic load and improve the efficiency of secondary processes [12]. The most acceptable approaches should be eco-friendly and non-polluting. Moreover, the removal techniques that are used should consider the types of pollutants and their concentration [13]. 

Adsorption is a widely accepted technique because it is easy to apply, efficient for pollutant removal, does not require supplements, and can be low cost when a natural absorbent is applied [13,14,15]. The adsorbent, a highly porous material, has a large surface area and many free functional groups that provide adequate and selective adsorption [16,17,18]. To ensure sustainability in wastewater treatment, for nearly two decades, natural sources have been studied as non-conventional adsorbent materials to remove or reduce pollutants in water, including dyes; they have demonstrated good adsorption capacity [19,20]. For example, Acid Blue 25 was removed using waste tea residue (50.8 mg g^−1^) [21]. Crystal violet and methylene blue were removed using sugarcane bagasse (107.5 and 112.9 mg g^−1^, respectively) [22]. Malachite green was removed by employing grape stalks (214.1 mg g^−1^) [23]. Methyl red was removed using eggshell powder (1.26 mg g^−1^) [24]. Cibacron blue was removed by utilizing pistachio shells (1.63 mg g^−1^ untreated and 4.53 mg g^−1^ chemically treated) [25]. Crystal violet was removed using activated carbon from pomegranate peels (23.26 mg g^−1^) [26]. Tartrazine was removed with activated carbon powder from cola nut shells (19.26 mg g^−1^) [27]. While these reports show that non-conventional adsorbents represent emerging and eco-friendly alternatives for water decontamination, most of them focused on the removal of only one dye. The purpose of the present study was to establish the adsorption capacity of the non-conventional natural and green material *Luffa cylindrica* (*Lc*) to remove an azo dye mixture.

## 2. Results

### 2.1. Azo Dye Mixture Removal

**Figure 1:**
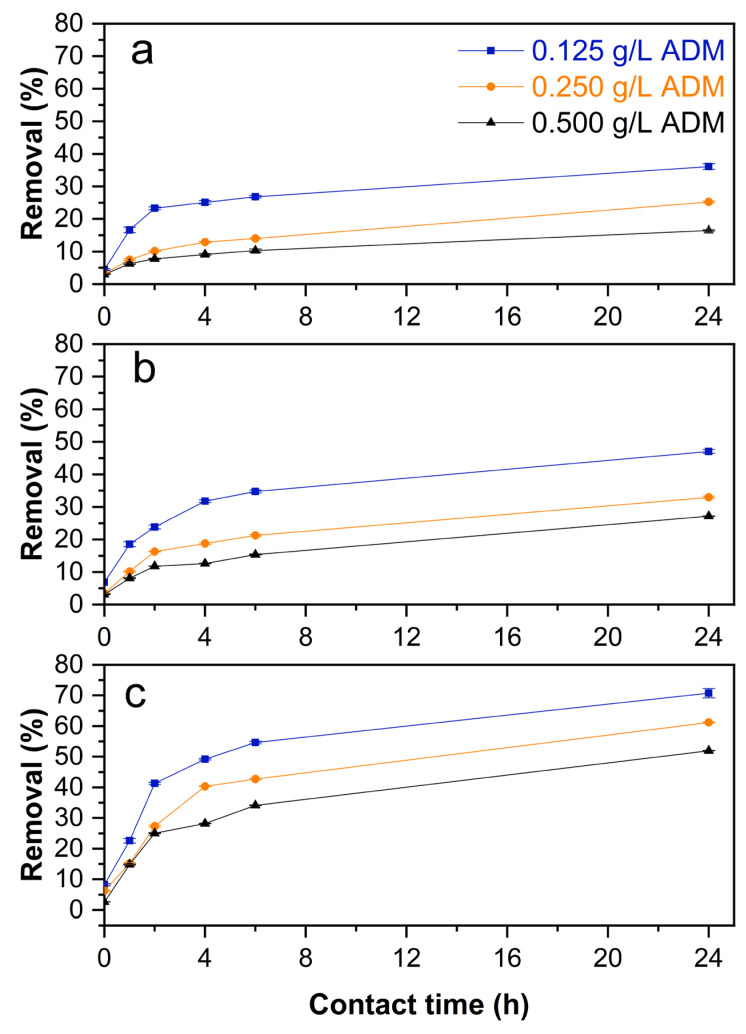
Removal of ADM using (**a**) 2.5; (**b**) 5.0; (**c**) 10 g L^−1^ of *Lc*.

Figure 1 shows the removal of ADM, considering 2.5, 5.0, and 10.0 g L^−1^ *Lc* doses.

### 2.2. The Effect of pH

**Figure 2:**
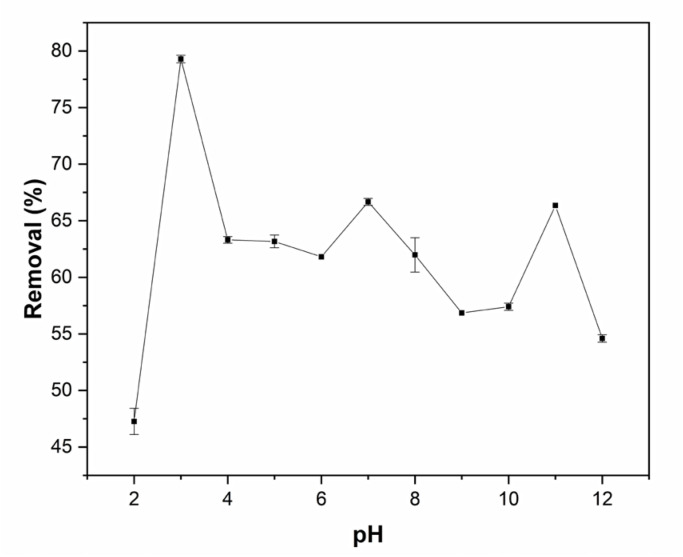
Effect of pH in ADM removal using *Lc* (adsorbent dose: 2.5 g/L, C_0_: 0.250 g L^−1^).

The effect of pH is shown in Figure 2. pH 2.0 led to the lowest ADM removal (47.3%); there was 60–70% ADM removal at pH 4.0–8.0. The highest removal (79.3%) occurred at pH 3.0. 

### 2.3. Adsorption Capacity

**Figure 3:**
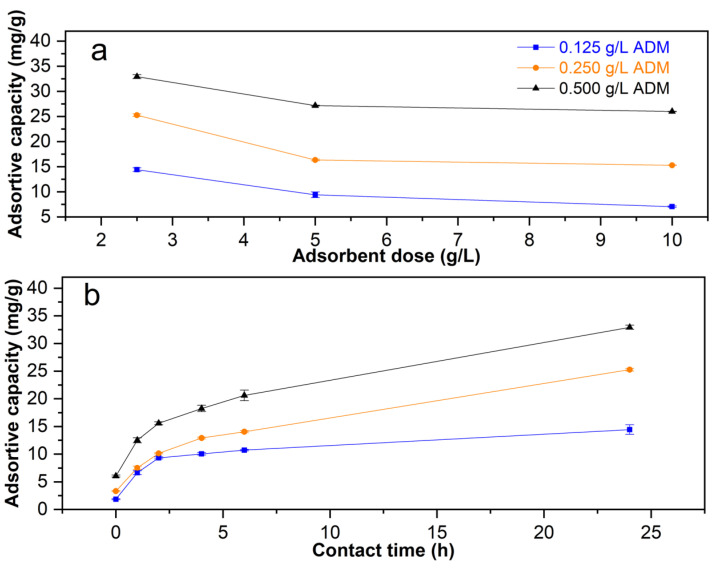
(**a**) Effect of the adsorbent dose and (**b**) effect of contact time on *Lc* adsorptive capacity (Q).

The adsorption capacity for 2.5, 5.0 and 10.0 g L^−1^ *Lc* dose is showing in Figure 3a. The time effect respect to adsotprion capacity for 2.5 g L^−1^ *Lc* is showing in Figure 3b. 

**Figure 4:**
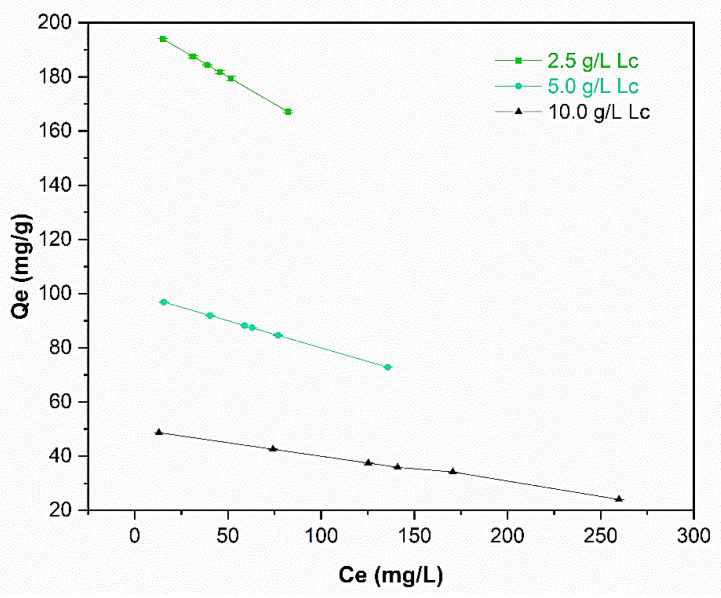
Concentration (C_e_) versus adsorptive capacity at the equilibrium (Q_e_).

The relation between concentration (C_e_) at the equilibrim and adsorption capacity (Q_e_), starting from 0.5 g L^−1^ ADM, are showing in Figure 4.

### 2.4. Adsorption Isotherms

**Figure 5:**
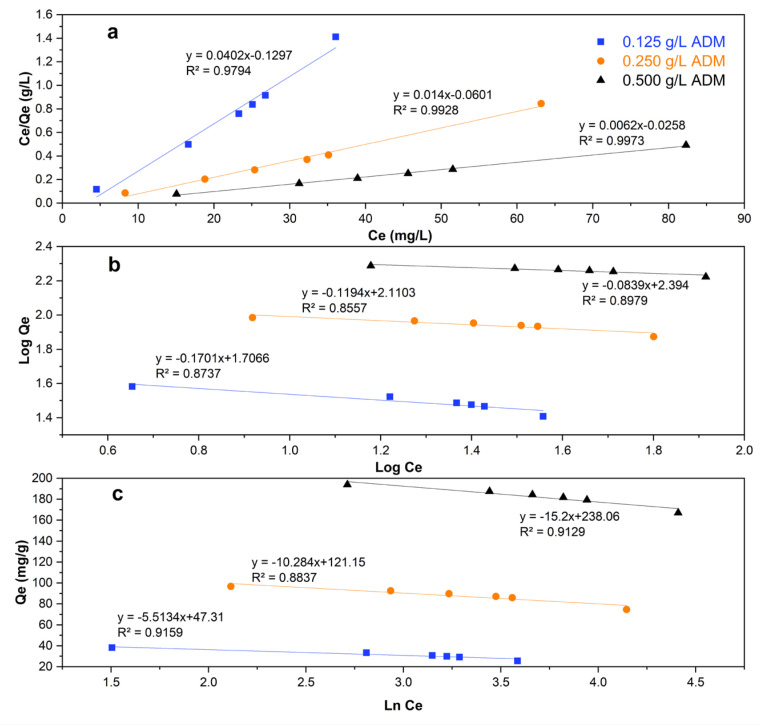
Langmuir (**a**), Freundlich (**b**), and Temkin (**c**) isotherm models for the adsorption of ADM (*Lc* dose: 2.5 g L^−1^).

Figure 5 shows how the adsorption isotherms fit the data. According to the correlation coefficient (R^2^) values, the Langmuir model (Figure 5a) provided the best fit. Thus, ADM adsorbs to the *Lc* surface by forming a homogeneous monolayer.

### 2.5. Adsorption Kinetics

**Figure 6:**
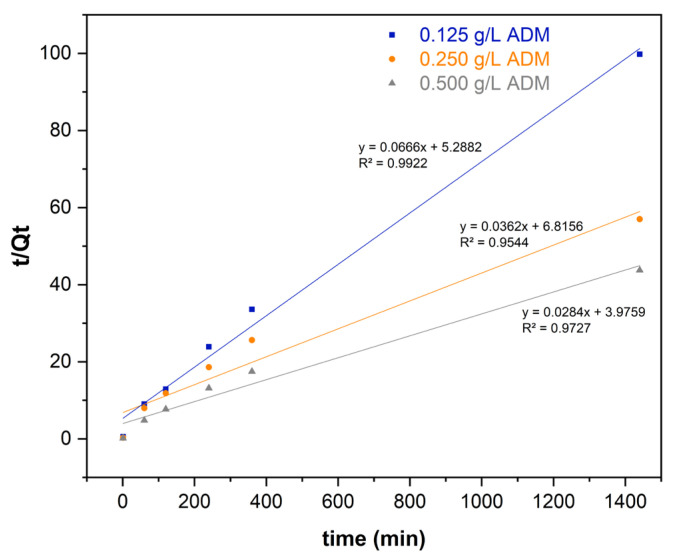
Pseudo-second order model for the kinetic adsorption of ADM (*Lc* dose: 2.5 g L^−1^).

The kinetic parameters from the models evaluated using 2.5 g L^−1^ *Lc* are presented in Table 1, and Figure 6 shows the best fit for the data. Table 2 compares the results from the present study with other adsorption studies using *Luffa* spp. to remove dyes in water [28,29,30,31,32,33]. 

### 2.6. FTIR Spectroscopy

**Figure 7:**
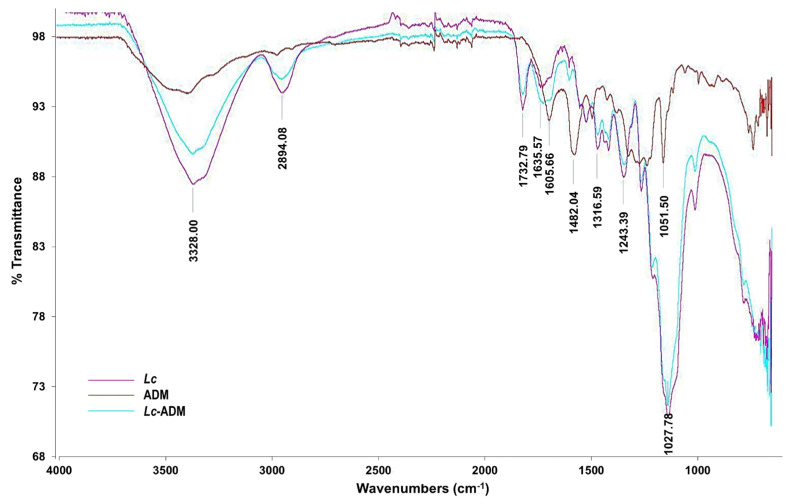
FTIR spectra of non-conventional and untreated *Lc* (pink line), ADM (reddish-brown line), and ADM adsorbed to *Lc* (blue-green line).

FTIR spectra (Figure 7) were acquired to analyze *Lc* with and without ADM and ADM only.

### 2.7. Morphology of Lc

**Figure 8:**
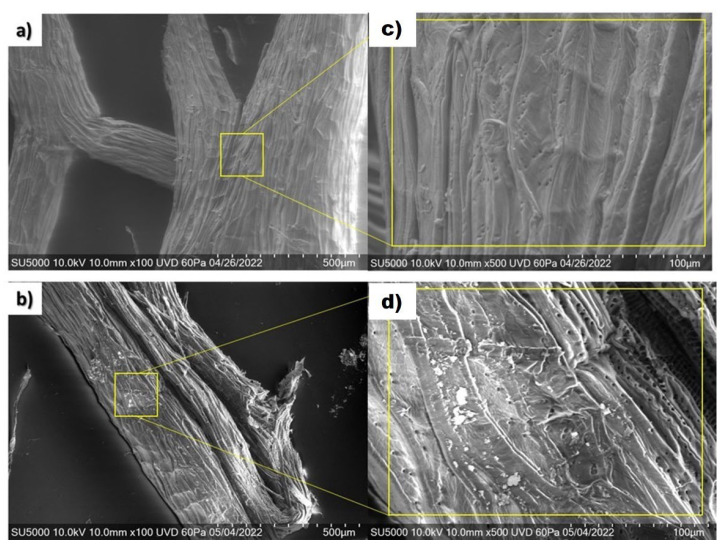
FESEM micrographs of (**a**) untreated natural *Lc*, (**b**) *Lc* after ADM adsorption, (**c**) amplification of untreated *Lc* (500), (**d**) amplification of *Lc* after ADM adsorption (500).

The analysis of FESEM micrographs of the *Lc* surface before and after ADM adsorption is shown in Figure 8.

Based on energy-dispersive X-ray spectroscopy analysis (EDXA), before adsorption, *Lc* (Figure 8c) was composed of 56.2% carbon and 38.2% oxygen (Table 3). 

## 3. Discussion

After 24 h as contact time, the highest *Lc* dose (10.0 g L^−1^) had removed 70.8% of ADM azo dye (line blue, Figure 1c). Based on previous studies, other lignocellulosic adsorbents could remove approximately 90% of dyes; however, most of these studies focused on only one dye or used chemically modified adsorbents [34,35,36].

pH is a crucial parameter in the adsorption process [37,38] because it impacts the binding sites on the adsorbent and alters the electronic environment of dissolved molecules. In acidic media, the surface is positively charged, and H^+^ ions compete with dye cations, meaning that less adsorbent interacts with the adsorbate [39,40]. On the other hand, in basic media, the lignin and cellulose chains of *Lc* fibers become negatively charged, and thus, the azo dyes cannot interact well with the adsorbent. Analysis of variance showed that pH significantly affects ADM removal (*p* < 0.05).

The adsorption capacity is enhanced at the lowest *Lc* dose and the highest ADM concentration (Figure 3a), and it increases with respect to time (Figure 3b). The highest Q_e_ was obtained when using 2.5 g L^−1^ *Lc*.

The adsorption of ADM along the entire surface of *Lc* is mediated by the formation of multiple, different, and strong physicochemical interactions like van der Waals forces [41]. Similar observations have been reported when using natural adsorbents from agricultural waste such as *Citrus limetta* peel [42], wheat straw [43], shaddock husk [44], and cottonseed husk [45], to mention a few examples, to remove dyes in solution. In each of the aforementioned studies, the Langmuir isotherm provided the best fit for the data. 

According to the R^2^ values (Table 1 and Figure 6), the pseudo-second-order model shows the best fit for the data. Hence, the removal process involves chemisorption [28]. In most of them, the Langmuir isotherm and the pseudo-second-order model best described the dye adsorption processes, even with other pollutants or adsorbents [14,46,47,48].

Unmodified *Lc* (green line, Figure 7) shows a broad absorption band at 3328 cm^−1^ characteristic of O–H. A broad shorter peak at 2894 cm^−1^ is attributable to C–H stretching vibrations. The bands at 1370, 1317, and 1243 cm^−1^ represent vibrations for –CH– and –CH_2_– groups [49]. The peaks at 1620 and 1424 cm^−1^ show conjugated double bonds. The strong peak at 1027 cm^−1^ is attributed to C–O–C and to C–O vibrations, bonds present in polysaccharides [50]. All these signals describe a lignocellulosic material [51]. The FTIR spectrum of ADM (blue line, Figure 7) shows the vibrations of the π conjugation system of C=C bending (1606 cm^−1^) and of N=N stretching (1482 cm^−1^), characteristics of amines. The sulfonate group vibrations are observed at 1396 cm^−1^ (the S=O double bond) and 1052 cm^−1^ (the S–O bond) [52]. A broad signal at 3354 cm^−1^ is attributed to N–H bonds for amines. The FTIR spectra of ADM adsorbed to *Lc* (reddish-brown line, Figure 7) show a broad signal at 3332 cm^−1^ assigned to N–H and O–H vibrations due to dye–adsorbent interactions. The azo group (–N=N) vibrates at 1580 cm^−1^, as confirmed by stretching vibrations at 1243 and 1158 cm^−1^. The signals at 1405, 1316, and 1031 cm^−1^ indicate the stretching vibrations for the sulfonate group present in the dye [53].

FTIR signals corresponding to hydroxyl, carbonyl, and alkyl functional groups, and aromatic vibrations are generally present in cellulose, hemicellulose, and lignin [54,55]. All of those vibrations are present in the *Lc*–ADM FTIR spectrum (reddish-brown line, Figure 7). Moreover, the peaks at 3328, 2894, and 1732–1482 cm^−1^ indicate that ADM involves stronger interactions with the hydroxyl and carbonyl groups of *Lc* and methylene hydrogens of the aromatic ring of ADM dye on the *Lc* surface. 

Figure 8c (enclosed zone in yellow) shows the fibrous structure of *Lc* with many small holes (0.5–4 μm in diameter). The smooth amorphous waxy/gummy layer indicates the presence of lignin [56]. Porosity increases the surface area of adsorbent material [17]. Figure 8d (enclosed zone in yellow) shows the changes in the surface of *Lc* after ADM adsorption. It is possible to observe that the holes are occupied by the ADM molecules. The large, brilliant zones could correspond to salts [42,57].

The data of composition *Lc* obtained by EDXA (Table 3) are consistent with the composition of cellulosic and hemi-cellulosic materials [15]. After adsorption (Figure 8d), *Lc* exhibited a composition of 53.4% carbon, 15.2% nitrogen, and 24.7% oxygen as the chief elements, along with trace amounts of other elements.

**Figure 9:**
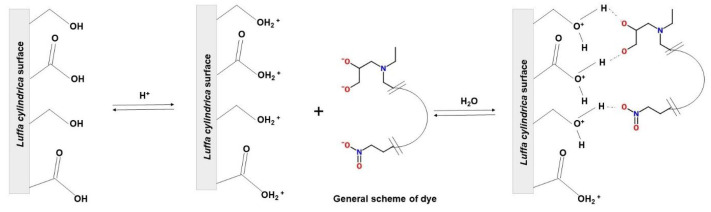
Mechanism of adsorption proposal to *Lc*-ADM in aqueous solution.

Thus, azo dye mixture likely adsorbs to *Lc* via hydrogen, electrostatic, chemical, and van der Waals interactions [54,58]. In the literature, researchers have reported various adsorption mechanisms depending on the chemical composition of the adsorbent, the nature of the pollutant, and the environmental media [11,18,59]. The adsorption process in solution involves water molecules forming hydrogen bonds between dyes and free functional groups on the *Lc* lignin–cellulose surface [60,61,62]. In agreement with the results of the isotherms and the FTIR spectra of the adsorbent after ADM adsorption, it is necessary to consider that the molecules and free ions play an important role in the adsorption process. Figure 9 shows a general mechanism for the *Lc*–ADM interaction to understand how adsorption occurs.

## 4. Materials and Methods

### 4.1. Luffa Cylindrica (Lc)

**Figure 10:**
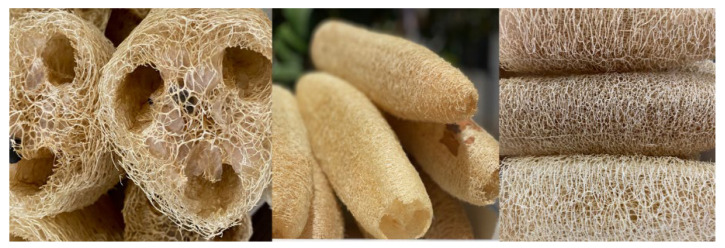
*Luffa cylindrica*; longitudinal and cross-sectional views of its structure.

*Lc* (Figure 10) is a fibrous, highly porous natural material composed of lignocellulose [13]. It has a large surface area with a large number of hydroxyl groups that can bind organic and inorganic molecules [63,64]. Dried *Lc* was acquired from Buenavista de Cuellar, Guerrero, Mexico (18°27′32″ N 99°24′20″ W). It was cleaned, the peel and seeds were removed, and it was cut into small 1 cm^3^ fragments, which were exhaustively washed using distilled water and dried using an oven (RIOSSA made in Mexico) at 40 °C for 24 h. These *Lc* fragments were used in the subsequent experiments as untreated natural fiber. 

### 4.2. Azo Dye Mixture

**Figure 11:**
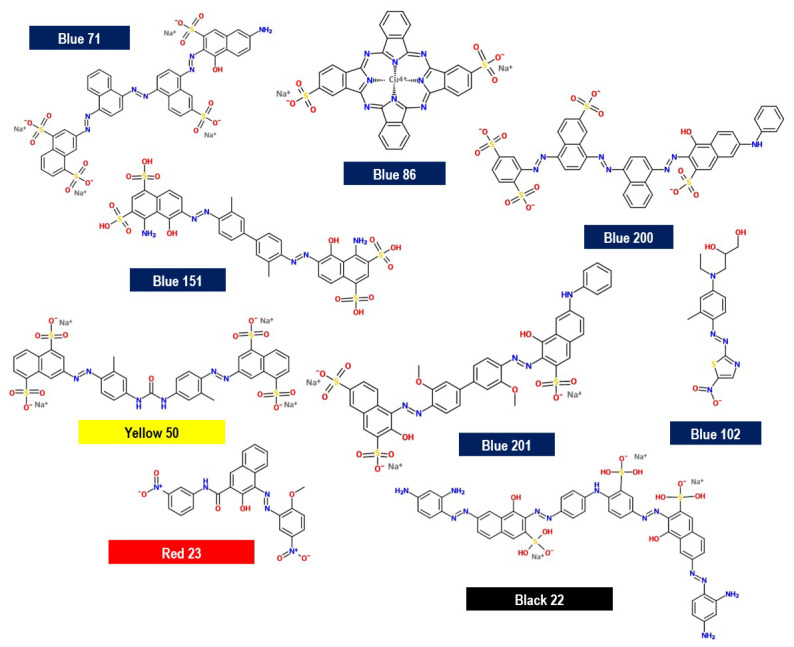
Chemical structure of dyes contained in ADM. Description: Monoazo dye: Disperse Blue 102 and Direct Red 23; Diazo dye: Direct Blue 151 and 201 and Direct Yellow 50; Triazo dye: Direct Blue 71 and Disperse Blue 200; Tetra-azo dye: Direct Black 22; and Phthalocyanine dye: Direct Blue 86.

A commercial mixture of azo dyes commonly used to renew the blue color of jeans at home was acquired from a local supermarket; the commercial mixture is commonly named ‘Indigo Blue’. Its formulation contains nine azo dyes: blue 71, 86, 102, 151, 200, and 201; black 22; red 23; and yellow 50 (Figure 11) as the major ingredients. They can be classified as mono-, di-, tri-, and tetra-azo dyes, one of which is a phthalocyanine class. This commercial azo dye mixture was renamed as ‘Azo dye mixture’ (ADM). A stock solution of ADM (1.0 g L^−1^) was prepared and used for all experiments. The maximum absorbance of ADM was determined using an ultraviolet–visible (UV–vis) spectrophotometer; it was detected at 550 nm. 

### 4.3. Experimental Design

In prior studies of adsorbents, researchers have generally used azo dye concentrations <0.100 g L^−1^ [28,29,30,65]. However, in real-world scenarios, dye concentrations are commonly >0.100 g L^−1^. For this study, it was necessary to establish a factorial experimental design for batch adsorption kinetic assays considering several azo dye and *Lc* doses (Table 4).

### 4.4. Adsorption Kinetics

The experiments were carried out in conical borosilicate glass flasks with a 50 mL working volume in static conditions at 28 ± 2 °C and pH 7.0; the contact time was 24 h. The residual ADM concentration was measured at 550 nm. All experiments were performed in quadruplicates, and the results were processed using Origin Pro-8 version 2018 and Minitab version 20 software.

The removal percent (R%) of ADM was calculated with Equation (1):

(1) $$R\%=\frac{C_{0}-C_{f}}{C_{0}}\times 100$$

where C_0_ is initial concentration, C_f_ is final concentration, and R% is the removal percent.

The adsorption kinetics was evaluated to understand ADM removal. The amount of ADM adsorbed by *Lc* (mg g^−1^) was calculated with Equation (2):

(2) $$Q=\frac{C_{0}-C_{f}}{m}\times V$$

where Q is the adsorption capacity (mg g^−1^), C_0_ and C_f_ are the initial and final concentrations (mg L^−1^), respectively, V is the volume (L) used, and m is the *Lc* mass (g) employed [28].

Several kinetic models were analyzed with the degree of adsorption controlled as a function of time. Equation (3) was used for the pseudo-first-order model:

(3) $$\ln\left(Q_{e}-Q_{t}\right)=\ln Q_{e}-k_{1}t$$

where Q_t_ is the ADM amount adsorbed by *Lc* at time t (mg g^−1^), and k_1_ is the adsorption rate constant (min^−1^).

The pseudo-second-order kinetic assumes that chemisorption is the rate-limiting step; it is represented in Equation (4) [34,42]:

(4) $$\frac{t}{Q_{t}}=\frac{1}{k_{2}{Q_{e}}^{2}}+\frac{t}{Q_{e}}$$

where t is the time (min), and k_2_ is the rate constant of pseudo-second order (g min mg^−1^).

Weber and Morris [66] proposed a theory based on interparticle diffusion and mentioned that adsorption occurs through a diffusion mechanism, as represented by Equation (5):

(5) $$Q_{t}=k_{id}t^{1/2}+C$$

where k_id_ is a rate constant of intraparticle diffusion (mg min^1/2^ g^−1^), and C is a dimensionless constant.

The Elovich model provides an explanation for adsorption of an adsorbate on the surface of an adsorbent. This model assumes that adsorbents have heterogeneous active sites with different active energies [67]. The model is represented in Equation (6):

(6) $$Q_{t}=\frac{\ln\left(\alpha \beta \right)}{\beta }+\frac{1}{\beta }\ln t$$

where α (mg g^−1^ min^−1^) is initial adsorption rate, β (g mg^−1^) is desorption constant, and t (min) is time [67,68].

### 4.5. Adsorption Isotherms

Adsorption equilibrium was analyzed to determine the adsorption capacity of *Lc* for ADM in aqueous solution. The amount of ADM adsorbed at equilibrium is expressed by Equation (7):

(7) $$Q_{e}=\frac{C_{0}-C_{e}}{m}\times V$$

where Q_e_ and C_e_ are the adsorption capacity and adsorbate concentration at equilibrium, respectively.

The type of adsorbent–adsorbate interaction, as well as the experimental conditions, is vital to determine which isotherm best fits the data. The equilibrium data were analyzed by considering the linearized Langmuir, Freundlich, and Temkin isotherms. The Langmuir isotherm has been commonly used to discuss various adsorbate–adsorbent combinations (liquid and gas phase adsorption) [29,30,65,69] and is represented by Equation (8):

(8) $$\frac{C_{e}}{Q_{e}}=\frac{1}{Q_{m}K_{L}}+\frac{1}{Q_{m}}C_{e}$$

where Q_e_ (mg g^−1^) is the adsorption capacity of the material at equilibrium; Q_m_ (mg g^−1^) is the maximum adsorption capacity to form a complete monolayer on the surface at the adsorbate concentration at equilibrium C_e_ (mg L^−1^); and K_L_ (L mg^−1^) is the Langmuir constant related to the affinity between the adsorbate and adsorbent [26]. 

The essential characteristics of the Langmuir isotherm model are known as the separation factor RL, which can be expressed by Equation (9):

(9) $$R_{L}=\frac{1}{1+K_{L}C_{0}}$$

If R_L_ is > 1, the adsorption is unfavorable. The adsorption is linear if R_L_ is 1. When R_L_ is >0 but <1, the absorption is favorable. Finally, if R_L_ is 0, then absorption is irreversible [31].

The Freundlich isotherm is represented by Equation (10):

(10) $$logQ_{e}=\frac{1}{n}logC_{e}+logK_{F}$$

where K_F_ is the Freundlich constant [(mg g^−1^) (L mg^−1^)^1/n^], and n is a dimensionless constant that is related to the absorption intensity of the adsorbent–adsorbate relationship, sometimes called the adsorption intensity or surface heterogeneity [26,70]. 

The Temkin isotherm is presented in Equation (11):

(11) $$Q_{e}=B*LnC_{e}+B*LnK_{T}$$

where B (J mol^−1^) is the Temkin constant, obtained from RT/b, where R is the universal gas constant (8.314 J mol^−1^), T is the temperature in Kelvin (298 °K), b is the variation in the absorption energy, and K_T_ is the Temkin equilibrium constant (L mg^−1^) [70]. 

### 4.6. Influence of pH on ADM Removal

The pH effect of ADM removal was evaluated. A solution of 0.500 g L^−1^ ADM was prepared using 0.01 M KNO_3_ as a buffer [28,30]. Then, the pH was adjusted between 2 and 12 by adding 0.1 M NaOH or HCl. *Lc* (2.5 g L^−1^) was added to each flask and incubated for 24 h. Samples were taken at 0 and 24 h and analyzed to calculate the percent ADM removal. 

### 4.7. Fourier-Transform Infrared (FTIR) Spectroscopy

FTIR spectra were obtained with a Spectrum One FTIR spectrophotometer (Perkin Elmer, Wellesley, MA, USA). The spectra included 128 scans from 500 to 4000 cm^−1^ at a resolution of 4 cm^−1^.

### 4.8. Field Emission Scanning Electron Microscopy (FESEM)

The internal three-dimensional (3D) morphology and the surface of *Lc* before and after dye adsorption were obtained with FESEM coupled to energy-dispersive X-ray spectroscopy using a Hitachi SU5000 microscope manufactured in Tokio, Japan. The fiber samples were sprayed with gold coating, and the analysis of samples was acquired by topographic contrast and registered at 100 and 500 magnification.

## 5. Conclusions

*Lc* could remove 70.8% of ADM dissolved in water in static conditions at 25 ± 2 °C, pH 7.0, and a contact time of 24 h. The adsorption capacity of *Lc* was 161.29 mg g^−1^; adsorption occurs according to the Langmuir isotherm, and the kinetics obeys the pseudo-second-order model. The statistical analysis showed that the adsorbent dose, the azo dye mixture concentration, pH, and contact time significantly influence azo dye mixture removal and the adsorption capacity. *Lc* is an eco-friendly and sustainable adsorbent. It acts as a non-conventional material to reduce azo dyes dissolved in water. It could be applied to coupled treatments to ensure pollutant concentrations are sufficiently reduced and that wastewater treatment performs efficiently.

## Figures and Tables

**Table 1:** Kinetic parameters of the adsorption of ADM using *Lc* (ADM = 0.125 g L^−1^, *Lc* = 2.5 g L^−1^, t = 24 h, T = 298 ± 2 °K; pH = 7.0).

Kinetic Model	Parameters	Value	R^2^
Pseudo-first order	k_1_ (min^−1^)	−0.0007	0.7974
	$Q_{e}$, observed	24.8756	
	$Q_{e}$, calculated	26.7384	
Pseudo-second order	k_2_ (g min mg^−1^)	0.0008	0.9922
	$Q_{e}$, calculated	15.0150	
Intraparticle diffusion	k_id_ (mg min^1/2^ g^−1^)	0.3085	0.8456
	C	4.0895	
Elovich	Β (g mg^−1^)	0.5967	0.9595

**Table 2:** Adsorption studies with *Luffa* spp. as adsorbent for dye removal.

Study	Adsorbent (g L^−1^)	Dye (mg L^−1^)	pH	T (°K)	t (h)	Q_m_ (mg g^−1^)	Isotherm Model	*k*_2_ (g mg^−1^min^−1^)	*R* ^2^	Reference
MG-*Lc*	0.05	20	5.0	308	5.0	29.40	L	0.013	0.996	[33]
MG-*Lap*	0.6	50	7.0	303	2.5	166.67	L	0.003	0.995	[31]
TB-*Lc*	1	10	7.0	303	0.5	45.60	L	0.0029	0.919	[29]
AB-*Lc*	1	20	2.0	293	4.0	9.63	F and T	0.004	0.999	[28]
MB-*Lc*	3	300	5.8	293	25.0	49.46	L	0.0051	0.999	[30]
MG-*Lap*	8	50	4.0	323	3.0	69.64	L	3.615	0.999	[32]
This study	2.5	125	7.0	298 ± 2	24	24.88	L	0.0008	0.992	This study
		250				71.43		0.0002	0.954	
		500				161.29		0.0002	0.973	

*Lc*: *Luffa cylindrical*; *Lap*: *Luffa aegyptica* peel; TB: Trypan Blue; AB: Alpacide Blue; MG: Malachite Green; MB: methylene blue; L: Langmuir; F: Freundlich; T: Temkin.

**Table 3:** Principal elements identified on the biomass surface by FESEM/EDXA.

Material	Element Content (%)					
	C	N	O	Na	Al	Si
Untreated *Lc*	56.2	N.D.	38.2	N.D.	5.2	0.4
*Lc*-ADM	53.4	15.2	24.7	0.8	4.6	0.4

N.D. No detected

**Table 4:** Experimental design.

Independent Variables	Level of Significance		
	Low (−1)	Medium (0)	High (+1)
*Lc* (g L^−1^)	2.5	5.0	10.0
ADM (g L^−1^)	0.125	0.250	0.500

## Data Availability

Data are contained within the article.
